# The HrpG/HrpX Regulon of Xanthomonads—An Insight to the Complexity of Regulation of Virulence Traits in Phytopathogenic Bacteria

**DOI:** 10.3390/microorganisms9010187

**Published:** 2021-01-16

**Authors:** Doron Teper, Sheo Shankar Pandey, Nian Wang

**Affiliations:** 1 Department of Plant Pathology and Weed Research, Agricultural Research Organization (ARO)-Volcani Center, Rishon LeZion 7505101, Israel; 2 Citrus Research and Education Center, Department of Microbiology and Cell Science, Institute of Food and Agricultural Sciences, University of Florida, Lake Alfred, FL 33850, USA; sshankar.pandey@ufl.edu

**Keywords:** *Xanthomonas*, HrpG, HrpX, type 3 secretion system, regulatory networks, phytopathogenic bacteria, transcriptional regulators

## Abstract

Bacteria of the genus *Xanthomonas* cause a wide variety of economically important diseases in most crops. The virulence of the majority of *Xanthomonas* spp. is dependent on secretion and translocation of effectors by the type 3 secretion system (T3SS) that is controlled by two master transcriptional regulators HrpG and HrpX. Since their discovery in the 1990s, the two regulators were the focal point of many studies aiming to decipher the regulatory network that controls pathogenicity in *Xanthomonas* bacteria. HrpG controls the expression of HrpX, which subsequently controls the expression of T3SS apparatus genes and effectors. The HrpG/HrpX regulon is activated *in planta* and subjected to tight metabolic and genetic regulation. In this review, we cover the advances made in understanding the regulatory networks that control and are controlled by the HrpG/HrpX regulon and their conservation between different *Xanthomonas* spp.

## 1. Introduction

*Xanthomonas* is a large genus of gamma-proteobacteria. *Xanthomonas* isolates are mostly plant-associated, and many are important plant pathogens that cause devastating effects on yield [1]. *Xanthomonas* species harbor extremely high host specificity and together infect hundreds of crops [1,2]. Because of their significant economic impact, *Xanthomonas* bacteria were extensively studied in the past three decades to achieve a mechanistic understanding of virulence functions and host specificity determinants. Most pathogenic *Xanthomonas* spp. facilitate pathogenic interactions with their hosts by secreting and delivering effector proteins into the host cell via the type 2 and 3 secretion systems [3]. Type 3 secretion system and secreted effectors are subjected to tight transcriptional regulation mediated by the two master regulators HrpG and HrpX. Here, we review the environmental factors and regulatory networks associated with virulence regulation in xanthomonads controlled by the HrpG/HrpX regulon.

## 2. Virulence and Occurrence of Secretion Systems in *Xanthomonas* spp.

*Xanthomonas* species encode six different protein secretion systems (types 1 to 6) and abundance, phylogenetic lineages, and the number of copies of each protein secretion system varies among species or pathotypes, and occurrence of all six secretion systems in a single species is common [2,4]. Type 6 and type 4 secretion systems (T4SS and T6SS) of various lineages are found in many species and have been suggested to play a role in the persistence of the bacteria in the environment. Chromosome-encoded T4SS of *Xanthomonas citri* subsp. *citri* (*Xcci*) was reported to provide the capacity of contact-dependent bacterial killing through the delivery of toxins to cells of neighboring bacteria [4], whereas T6SS was identified to prevent predation by the soil amoeba through an unknown mechanism [5]. Most *Xanthomonas* strains harbor two type 2 secretion systems (T2SS) classified as *xcs* and *xps.* The contribution of the *xcs* T2SS to pathogenicity on plants or persistence in the environment is poorly understood, and the system is absent in several *Xanthomonas* species such as *Xanthomonas oryzae* pv. *oryzae* (*Xoo*) [2]. The *xps* T2SS is conserved in the genomes of all sequenced *Xanthomonas* species and is required for full virulence of *Xoo*, *Xanthomonas euvesciatoria* (*Xeu*), and *Xcci* [6,7,8]. The *xps* T2SS enables the secretion of numerous secreted hydrolases [9]. Secreted hydrolases such as xylanase, cellulases, esterases, and pectinases function as virulence factors in many *Xanthomonas* species and are required for full virulence on host plants [9,10,11,12]. However, cell wall hydrolysis by these hydrolases can also elicit immune responses through host recognition of damage-associated molecular patterns [13,14].

**Figure 1:**
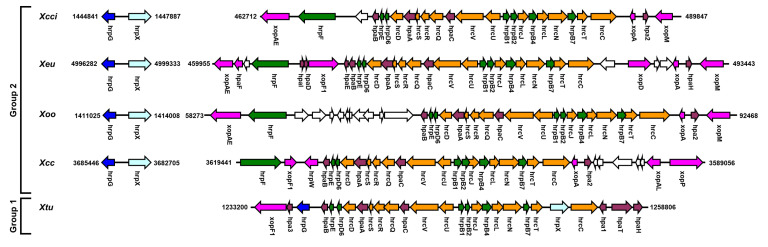
Genetic organization of the hypersensitive reaction and pathogenicity (*hrp*) gene cluster and the *hrpG/hrpX* coding regions in *Xanthomonas* spp. Schematic representation of the gene clusters of group 2 xanthomonads *X*. *citri* subsp. *citri* (*Xcci,* strain 306), *X. euvesicatoria* (*Xeu*, strain 85-10), *X. oryzae* pv. *oryzae* (*Xoo*, strain KACC10331), and *X. campestris* pv. *campestris* (*Xcc*, strain 8004), and group 1 Xanthomonas *X*. *translucens* pv. *undulosa* (*Xtu*, strain 4699). The location of the indicated regions within their respective genomes is stated.

Flagellar and injectisome type 3 secretion systems are abundant among *Xanthomonas* species [3]. The flagellar T3SS (FT3SS) is required for motility, biofilm formation, and chemotactic movement [15,16,17]. Disruption of the FT3SS affects entry and infectivity of the bacteria and is mostly associated with the epiphytic stage of *Xanthomonas* life cycle [1,17]. Injectisome type 3 secretion system (T3SS) enables the translocation of effector proteins from bacteria to eukaryotic cells which affect host signaling and metabolism [18]. Many bacteria utilize this system to establish pathogenic or symbiotic interactions with their eukaryotic hosts. A hypersensitive reaction and pathogenicity 2 (*hrp2*) family injectisome T3SS [19,20] is found in the majority of pathogenic *Xanthomonas* strains and is considered as one of the main pathogenicity factors of the bacteria [3,21]. Almost all pathogenic group 2 *Xanthomonas* species harbor T3SS genes that are localized in a ~30 kb chromosomal pathogenicity island and composed of approximately 20 core structural component coding genes in six transcriptional units (A to F), while additional T3SS accessory and effector coding genes within the pathogenicity island vary between species or pathotypes [22] (Figure 1). Disruption of any key structural component of the T3SS results in the inability to cause disease, colonize host plant, and induce hypersensitive response (HR) in resistant host varieties [3]. In addition, *hrp*-dependent pathogenic *Xanthomonas* strains encode on average 25–35 T3SS effectors (T3Es) that were reported to function in manipulating host immune signaling, liquid accumulation in the intercellular spaces, and nutrient accessibility [2,3,21]. The biochemical properties, host targets, and functions of T3Es were previously reviewed [2,3,21]. Several group 2 xanthomonads, such as *X. maliensis, X. cannabis*, and multiple *X. arboricola* strains that lack T3SS or harbor a reduced arsenal of T3Es were identified in the past decade [23,24,25]. These bacteria display reduced growth *in planta*, do not cause symptoms in host plants, and are considered as commensal strains. A direct correlation between pathogenicity to the occurrence of *hrp2* T3SS in group 1 xanthomonads is less apparent and appears to be species-specific; the T3SS is required for full pathogenicity of *X. translucens* strains but is absent in sugarcane pathogenic bacteria *X. albilineans* and *X. sacchari* [1]. In addition, the T3SS genomic islands of group 1 xanthomonads share low homology and altered gene composition compared with group 2 xanthomonads, indicating that T3SS was acquired independently in these strains [26] (Figure 1). For instance, the *hrpF/nolX* gene that encodes the T3SS translocon in group 2 xanthomonads is absent in group 1 that harbors an alternative translocon coding gene, *traT* [27].

## 3. HrpG and HrpX Are Key Virulence Regulators in *Xanthomonas* spp.

The ability to cause disease in host plants in *hrp*-dependent *Xanthomonas* spp. is dependent on two transcriptional regulators—HrpX and HrpG [3]. HrpX was first identified in the early 1990s in a transposon screen in *X. campestris* pv. *campestris* (*Xcc*) [28] and soon after was found to have similar functions in other *Xanthomonas* spp. [29,30]. The *hrpX* gene encodes a ~476 amino acids (AA) (sizes may vary between species, mostly due to alternative prediction of the start codon) AraC-type bacterial transcriptional regulator [31]. Disruption of *hrpX* in *Xanthomonas* bacteria that harbor T3SS completely abolished the ability to cause disease, colonize the plant host, or induce HR in resistant or non-host plants in a similar manner to disruption of structural components of the T3SS [3]. Further analyses demonstrated that transcriptional regulation of the six T3SS transcriptional operons and multiple T3Es is dependent on HrpX [22,30,32,33,34], thus establishing HrpX as a master regulator of *hrp*-dependent protein secretion.

**Figure 2:**
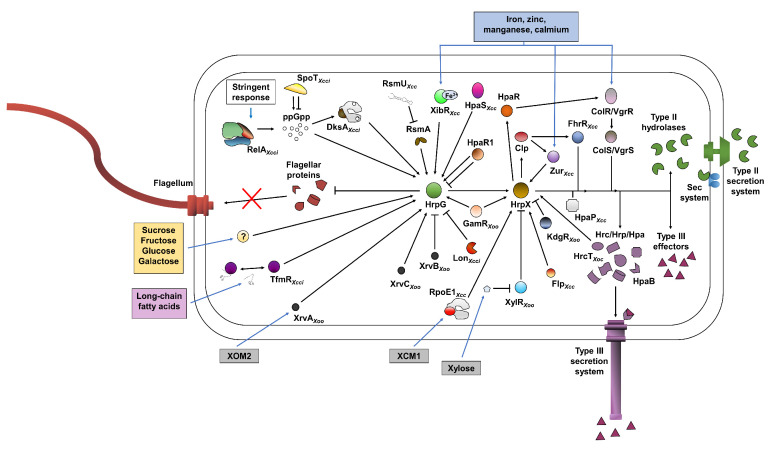
Schematic representation of the metabolic and genetic regulation of *hrpG* and *hrpX* in *Xanthomonas*. Metabolites and environmental cues are represented in rectangles. Arrows and “⊣” signs indicate that a protein promotes or inhibits the target, respectively, based on transcriptional or functional analyses. If the analysis was based on data derived from specific *Xanthomonas* species, it is marked at the bottom of the represented protein names. Data represent information derived from *X. euvesicatoria*, *X*. *citri* subsp. *citri* (*Xcci*), *X. oryzae* pv. *oryzae* (*Xoo*), *X. oryzae* pv. *oryzicola* (*Xoc*), *X. axonopodis* pv. *glycines* and *X. campestris* pv. *campestris* (*Xcc*).

HrpG was identified in a screen for virulence-associated mutants using N-methyl-N′-nitro-N-nitrosoguanidine mutagenesis in *Xeu* [35]. Similarly to *hrpX*, inactivation or deletion of *hrpG* eliminates the ability of the bacteria to induce HR in resistant or non-host plants, or cause disease and colonize host plants [35,36,37]. *hrpG* inactivation mutants also display a significant reduction in the transcription of T3SS and T3Es coding genes and *hrpX* itself [35,36,37] (Figure 2). *In vitro* studies showed that HrpG directly binds to the promoter region of *hrpX* and induces the transcription of *hrpX*, indicating that *hrpX* is a direct target of HrpG [38,39] (Figure 2). *hrpG* encodes a ~260–267 AA orphan OmpR family transcription regulator [35]. While HrpG structure was not experimentally determined, the mechanistic function was characterized by several key point mutations that affected its activity. Most OmpR regulators are part of a two-component system and are encoded in an operon or in the proximity of a histidine kinase that activates OmpR protein by phosphorylation [40]. HrpG harbors a conserved aspartate residue at position 60 (of *Xcci* and *Xeu* HrpG) that serves as a phosphorylation site; however, no histidine kinase gene is encoded in proximity to *hrpG*. HrpG D60N substitution mutant of *Xcci* is not phosphorylated in *hrp*-inducing conditions and cannot complement the *hrpG* mutant [41], suggesting that HrpG is activated through phosphorylation by an unknown kinase. The mutation that resulted in R210C substitution in *Xcci* HrpG produced a dominantly negative inactive variant [38]. This mutant variant was not able to complement the *hrpG* inactivation mutant and reduced the expression levels of HrpG associated genes and effector delivery when introduced into wild-type *Xcci* [38]. *In vitro* assays identified that this HrpG variant can still bind to the *hrpX* promoter but is unable to induce the transcription of *hrpX* [38]. Random mutagenesis conducted in *Xeu hrpG* identified three constitutively active variants (corresponding to AA substitution of E44K, H194R, D199N), which induced the expression of *hrp* genes in non-inducing media and caused enhanced HR in resistant plants [42]. The mechanism that enables the constitutive activity by these variants remains elusive.

## 4. Genomic Organization of *hrpG* and *hrpX*

*hrpG* and *hrpX* are found in all reported pathogenic *Xanthomonas* spp. with the exception of *X. albilineans* and *X. sacchari* that lack the *hrp2* T3SS gene cluster [1], and they are regarded as the master regulators of the T3SS system in xanthomonads [3]. Homologs of these two regulators were found to function in a similar manner in phytopathogenic bacteria such as *Ralstonia solanacearum*, *Burkholderia Pseudomallei,* and *Acidovorax citruli* [19,43,44,45,46]. In group 2 *Xanthomonas* spp., *hrpG* and *hrpX* are localized in the same genomic region in a reverse orientation of one another spaced with a ~760–840 intragenic region (Figure 1 and Figure S1). While *hrpG* and *hrpX* share high DNA sequence similarity between different species, the *hrpG*–*hrpX* intragenic regions that contain their promoter elements vary between xanthomonads (Figure S1). For instance, the *hrpG* and *hrpX* coding regions of *Xeu* and *Xoo* share 87% and 91% DNA sequence identity, respectively (Figure S1). On the other hand, the *hrpG*–*hrpX* intragenic regions of *Xeu* and *Xoo* share 67% DNA sequence identity (Figure S1). The variations in the *hrpG*–*hrpX* intragenic regions suggest that *hrpX* and *hrpG* might be subjected to differential regulation in different *Xanthomonas* species.

In *Xanthomonas* spp. of group 1, *hrpG* and *hrpX* are located in the *hrp2* T3SS genomic island [24] (Figure 1). *hrpG* is localized at the end of the *hrp2* gene cluster, whereas *hrpX* is in an operon with *hrcC* that encodes the structural ring protein of the T3SS basal body [47]. As of now, besides the knowledge that *hrpG* is somewhat required for the virulence of group 1 *X. translucens* [47], there’s no information regarding the upstream or downstream regulatory networks that control group 1 xanthomonads *hrpG* and *hrpX*. Therefore, all the information available in this review will only cover the HrpG/HrpX regulatory network of group 2 xanthomonads.

## 5. Downstream Targets and Regulatory Pathways Controlled by HrpG and HrpX

HrpG and HrpX are master regulators of *Xanthomonas* T3SS and T3E. HrpG-mediated expression of most of T3SS and T3Es genes is dependent on *hrpX*, which is directly regulated by HrpG [35,36]. HrpX induces transcription by directly binding to plant-inducible promoter (PIP) box, a DNA motif of TTCGB-N15/N8-TTCGB found in *cis* element of five of the six structural T3SS operons and multiple T3Es [22]. The DNA-binding motif of HrpG remained elusive for years. A recent study [39] utilized ChIP-seq analysis to identify HrpG*_Xcc_*-bound promoter sequences. The study found that HrpG physically interacted with 186 promoter elements. Promoter analysis identified a common DNA motif of ATT(C/T)(C/T)(G/C/A)(T/A)T in the bound promoters and demonstrated direct binding *in vitro*. It should be noted that the study failed to demonstrate a robust correlation between the HrpG bound promoters and transcriptional induction of these targets by HrpG [39]. For instance, *hrpX*, which is hypothesized to be one of the main direct targets of HrpG, was not among the 186 promoters.

Functional studies found that accumulation of active HrpG and HrpX proteins is the main factor that controls the expression of the T3SS, and constitutive expression of either of these regulators results in the induction of the T3SS and T3E in non-inducing media, hypervirulence upon inoculation of susceptible plants and enhanced immune responses in resistant plants [3]. It was recently suggested that the regulatory network of the T3SS harbors an internal positive feedback loop in *X. oryzae* pv. *oryzicola* (*Xoc*), in which *hrpX* expression is regulated by T3SS protein HrcT [48] (Figure 2). It is unknown whether this mechanism is unique to this species or it is conserved among *Xanthomonas* spp.

High throughput transcriptional profiling analyses of the HrpG regulon and/or the HrpX regulon were conducted in *Xeu*, *Xcci,* and *X. campestris* pv. *raphani* (*Xcr*) [36,37,49,50]. Two independent transcriptome studies (conducted using microarray and RNA-seq) were done with *hrpG* and *hrpX* deletion strains of *Xcci* grown in plant-mimicking media [37,49]. Transcriptomic studies of *Xeu* and *Xcr* that were grown in rich media compared the wild-type strain with a strain harboring the constitutively active HrpG E44K variant (hrpG*) using cDNA-AFLP [36] and RNA-seq [50], respectively. T3SS and T3E coding genes were found to be positively regulated by HrpX or HrpG in all four studies [36,37,49,50] (Figure 2). The T3SS gene *hrcC* and multiple T3E genes that lack plant-inducible promoter (PIP) box in their promoters were induced by HrpX and HrpG as well. However, the expression of several T3Es was not HrpG/HrpX dependent. In particular, HrpG and HrpX did not affect the expression of all four transcription activator-like effectors (TALEs) coding genes in *Xcci* [37,49]. Unlike most other T3Es, which were identified to be associated with suppression of host immune responses, TALEs were found to control more diverse factors such as sugar efflux and cell identity [51]. It is possible that TALEs are regulated independently of the rest of the T3Es because of their unique functions.

In addition to T3SS-associated genes, the four transcriptome analyses identified that HrpG or HrpX positively regulates a large number of genes encoding secreted hydrolases, such as endoglucanases, proteases, esterases, and pectinases [12,36,37,49,50] (Figure 2). Unlike the T3SS, the operons that encode structural components of either of the two T2SS identified in most *Xanthomonas* spp. were not regulated by HrpG or HrpX. Interestingly, several studies reported that HrpG or HrpX negatively regulates secreted protease activity even though a large number of genes encoding secreted peptidases are positively regulated by the two regulators [36,52,53]. The inconsistencies between the reported transcriptional regulations and functional activity need to be addressed.

The transcriptome analysis of *Xcci* identified that multiple genes associated with flagellar assembly and chemotaxis were upregulated in the *hrpG* mutant [37] (Figure 2). Supporting the transcriptional data, the *hrpG* mutant demonstrated increased motility in soft agar plates [37]. The transcriptional expression of these genes was not affected in the *hrpX* mutant, suggesting that HrpG negatively regulates these genes independently of HrpX [37]. The bacterial flagella are recognized by the host immune system and therefore the association of flagellar shedding with bacterial virulence signaling seems reasonable [54]. However, the mechanism behind HrpG regulation of flagellar assembly and motility remains poorly understood.

HrpG and HrpX were reported to induce the expression of *hpaR*, which encodes a MarR family transcriptional regulator in multiple bacteria [36,37,49,50] (Figure 2). MarR proteins are ubiquitous bacterial transcriptional regulators that repress or activate transcription by binding to small ligand molecules that alter their binding affinity to operator sequences found in the promoters of their target genes [55]. Proteins of the MarR family were identified to regulate metabolic processes, antibiotic resistance, and stress responses [55,56]. The *hpaR_Xcc_* deletion mutant was unable to colonize host plants or induce HR response in resistant plants [53]. Further analysis found that HpaR*_Xcc_* positively regulates the ColS/ColR (VgrS/VgrR) two-component system [57] that was independently identified to positively control *hrp* gene expression through an unknown mechanism [58,59,60].

## 6. Metabolic Regulation of the HrpG/HrpX Regulon

The expression and protein accumulation of HrpG and HrpX are highly dependent on the environment and the metabolic state of the bacterium. The two regulators are strongly expressed upon reaching the plant apoplast [61,62,63,64,65]. By utilizing GFP promoter fusions, Zhang et al. [61] demonstrated that *hrpG*, *hrpX*, and T3SS translocator *hrpF* of *Xeu* are specifically induced upon entering tomato substomatal cavities, whereas their expression was repressed on the leaf surface.

The transcriptional expression of *hrpG*, *hrpX*, and their target genes *in planta* was examined in numerous studies which usually focused on a time frame of 24–48 h post-inoculation [61,62,63,64,65]. However, several direct and indirect studies indicates that *hrp* expression starts much earlier, if not instantly upon the introduction of the bacteria into the plant environment. de Bernonville et al. have shown that *hrpE* is highly induced in *Xcc* 4 h after inoculation of cabbage [64]. In addition, effector delivery monitored through CyaA reporter fusions or transcriptional expression of TALE-induced plant susceptibility gene suggests that effectors can be actively delivered by the T3SS into the host cells between 12–24 h post-inoculation [66,67].

Induction of the *hrpG/hrpX* regulon is mediated through sensing the plant environment. Supplementing minimal media with rice extract or cabbage xylem sap almost immediately promoted the upregulation of *hrpG* and *hrpX* in *Xoo* and *Xcc* [64,68]. Undefined rich media such as NB and LB suppress *hrp* gene expression, whereas several *hrp*-inducing plant-mimicking artificial media were found to induce *hrp* gene expression. These defined media are of slightly acidic pH supplemented with trace metals, amino acids, and low molecular weight carbon sources such as mono- and di-saccharides or carbonic acids. Species specific responses were identified in different types of media: XVM1 and XVM2 media,, which are based on fructose and sucrose as a carbon source and methionine (for XVM1) or casamino acids (for XVM2) as a nitrogen source, induces *hrp* gene expression in pepper and citrus pathogens *Xeu* and *Xcci* [37,69]. XOM2, which uses xylose as a carbon source and methionine as the sole supplemented amino acid, promotes *hrp* expression in the rice pathogen *Xoo* [70]. MME and XCM2 media, which use either glutamate or succinate as the main carbon source and are supplemented with a low abundance of casamino acids and magnesium (in XCM2), were found to be effective for *hrp* expression in *Xcc* [71]. The differential responses of different *Xanthomonas* bacteria to different media suggest that, while the key regulatory elements of the T3SS are conserved, the metabolic regulation of the *hrp* is different in *Xanthomonas* spp. This differential regulation is possibly linked with the unique niche occupied by each species. It should be noted that discrepancies between the transcriptional responses of *Xanthomonas* to artificial plant-mimicking media and transcriptional responses within the plant environment were observed. For instance, GntR-family transcriptional regulator HpaR1/YtrA of *Xcc* and *Xcci* negatively regulates the T3SS associated genes in plant-mimicking media while positively regulates their expression *in planta* [63,72] (Figure 2).

The metabolic regulation of *hrp* gene expression is multilayered and integrates multiple environmental factors. The repression of *hrp* gene expression by rich undefined media suggests that the response is repressed by certain metabolites or activated by starvation. Stringent response in bacteria is induced by stress or metabolite starvation [73] and is mediated by internal levels of guanosine pentaphosphate [(p)ppGpp], which is synthesized and degraded by RelA and SpoT [73]. It was recently reported that deletion of *relA* and *spoT* homologs in *Xcci* completely abolished pathogenicity in citrus and caused a significant reduction in the expression of *hrpG, hrpX,* and their downstream targets [74]. Similar phenotypes were observed upon deletion of transcriptional regulator *dksA* [74], which is associated with alteration of the affinity of the RNA polymerase to (p)ppGpp [75] (Figure 2). These observations suggest that *hrp* gene expression is regulated by internal levels of (p)ppGpp that are presumably controlled by nutrient limitation. Most plant-mimicking media induce *hrp* gene expression to a significantly higher level compared with minimal media such as M9, which is more limited in nutrients [69]. Taken together, it seems that starvation-induced stringent response is not sufficient to induce the HrpG/HrpX regulon.

Carbon sensing plays an essential role in the activation or suppression of the *hrp* regulon. Utilization of sucrose and fructose as the main carbon source strongly induces *hrp* gene expression in *Xeu*, whereas such genes are not responsive to other sugars such as xylose and mannitol [69]. On the other hand, xylose appears to be a key inducer of *hrp* gene expression in *Xoo* [70]. Further studies have identified that the contribution of xylose to *hrp* gene expression in *Xoo* occurs downstream of the transcriptional regulation of *hrpX*. Ikawa and Tsuge reported that sucrose, galactose, fructose, and glucose promote the up-regulation of *hrpG* and *hrpX* [76,77], whereas structural components of the T3SS are transcriptionally upregulated specifically in the presence of xylose, presumably by stabilizing the HrpX protein [76] (Figure 2). Accordingly, deletion of the xylose responsive LacI-like transcriptional repressor *xylR* resulted in higher accumulation of HrpX protein and enhanced expression of T3SS structural genes as well [78] (Figure 2). The Clp/CRP transcriptional regulator is associated with cAMP-mediated carbon regulation in proteobacteria [79]. Disruption of *clp* in several *Xanthomonas* spp. abolished virulence and exoenzyme production [80,81,82,83]. A few studies suggested that Clp is indirectly associated with *hrp* gene regulation. For instance, *Xcc clp* mutant displays reduced expression of several effectors and *hrp* genes through the TetR transcriptional regulator FhrR [81] (Figure 2). A *fhrR* homolog is absent in many *Xanthomonas* species, such as *Xeu*, *Xoo*, *Xcci,* and *X. citri* pv. *glycines* (*Xcg*). Indeed, a second transcriptome-based study conducted in *Xcg* did not identify *Clp*-dependent *hrp* regulation [83]. However, the study found that *clp* is regulated by HrpG and HrpX in *Xcg*, further establishing that Clp and Hrp regulation are somewhat interconnected [83] (Figure 2).

Negative metabolic regulation by specific carbonic compounds was reported in numerous studies. Supplementation of XVM1 with TCA cycle-associated carbonic acids citrate, succinate, and pyruvate represses *hrp* gene expression in *Xeu* [69]. Exogenous supplementation of long-chain fatty acids such as oleic and myristic acids represses *hrp* gene expression in *Xcci* [84]. This suppression was linked to the TetR-family transcriptional repressor TfmR that directly regulates fatty acid beta-oxidation genes in response to long-chain fatty acids [84] (Figure 2). Accordingly, disruption of *tfmR* resulted in significantly reduced virulence in citrus plants that can be partially complemented by overexpression of *hrpG* [84].

## 7. Influence of Iron and Other Metals on HrpG/HrpX Regulation

Iron sensing, uptake, and the maintenance of intracellular iron homeostasis play a crucial part in xanthomonads virulence [85,86,87,88,89,90]. Multiple evidence supports that iron starvation plays a significant role in the regulation of the HrpG/HrpX regulon inside the host. Microarray-based transcriptome analysis showed induced expression of multiple *hrp* genes, including *hrpG* and *hrpX*, of *Xcc* under low iron conditions [91]. Further promoter probe analyses using GUS reporter fusions demonstrated upregulation of *hrp* gene cluster and *hrpG* in *Xcc* under iron depletion [90]. Supplementation of exogenous iron to the *hrp*-inducing media XCM2 drastically suppresses the expression of *hrpG* and the *hrp* gene cluster in *Xcc* [71,90]. Accordingly, external supplementation of iron suppresses the HR induction on tomato leaves by *Xcc* and *Xoc* [71,90]. However, high iron availability does not affect HR induction by *Xoo* [90]. In addition, *Xcc* also displayed a drastic reduction in virulence on cabbage leaves under iron-replete condition, indicating that the pathogenicity of *Xcc* is activated by iron depletion [90].

Regulation of iron and metal homeostasis is multilayered and harbors several key regulators. In addition to responding to excess/depletion of iron and other metals, these regulators were shown to control many cellular functions and traits associated with pathogenicity. XibR, an orphan response regulator, binds with ferric iron and regulates several virulence-associated functions, including iron uptake and metabolism, T3SS and T3Es expression, chemotaxis, and motility in response to iron availability in *Xcc* [91]. Deletion mutant of *xibR* in *Xcc* displays a substantial reduction in both *in planta* colonization and virulence on cabbage [91] (Figure 2). In contrast, Ferric Uptake Regulator (Fur) binds with ferrous iron and regulates multiple cellular functions including iron homeostasis [92]. *fur* mutant of *Xoo* showed hypersensitivity to oxidative stress and reduced virulence on rice plants [85]. The Zinc Uptake Regulator (Zur) transcriptional regulator is involved in the suppression of zinc uptake to maintain cellular zinc homeostasis. This regulator was also found to indirectly upregulate *hrpX* expression in *Xcc* under low iron conditions [93] (Figure 2). The ColS/ColR (VgrS/VgrR) two-component system was reported to play a critical role in iron and metal homeostasis and virulence regulation of multiple *Xanthomonas* spp. [58,59,60]. The ColS/ColR system is essential for pathogenicity and disruption of this system eliminates the ability to colonize the host and cause disease [58,59,60]. The ColS/ColR system was identified to respond to the availability of iron, zinc, manganese, and cadmium and was found to be critical for survival in excess/depletion of iron and other metals [58,59,94]. ColS is a membrane-bound receptor histidine kinase that senses extracytoplasmic iron limitation in the periplasm, while its cognate response regulator ColR detects intracellular iron excess [94]. The ColS/ColR system controls many cellular processes and pathogenicity-associated traits, such as biofilm formation, lipopolysaccharide production, catalase activity, iron uptake, tolerance to environmental stresses, and *hrp* gene expression [59,60,94] (Figure 2). Therefore, its effect on bacterial pathogenesis is multilayered and does not seem to be a result of an alteration of one particular trait. Apart from indirect transcriptional control of *hrp* and T3E genes, the interaction of the ColS/ColR system with the *HrpG/HrpX* regulon is not fully understood. The ColS sensor does not interact or directly induce the phosphorylation of HrpG and ColR-mediated interaction was not identified with the promoter sequences of *hrpG* or *hrpX*. The transcriptional regulator HpaR, which is positively regulated by HrpG/HrpX, was identified to directly control the *colS/colR* operon [57] (Figure 2). This indicates a cross-talk occurs between the *col* and the *hrp* regulons. However, such interaction still cannot fully explain downstream regulation of *hrp* genes by the ColS/ColR system and further study should be dedicated to the understanding of the regulatory network that connects these two regulons.

## 8. Species-Specific Control of the HrpG/HrpX Regulon by Diffusible Signal Factor

Diffusible signal factor (DSF) is a *cis*-unsaturated fatty acid that serves as a cell to cell signal molecule that is considered as the main quorum-sensing signal in *Xanthomonadales* [95]. DSF was reported to play a role in the virulence of many *Xanthomonas* bacteria by controlling multiple traits, including biosynthesis and dispersion of exopolysaccharides (EPS), motility, siderophore production, biofilm formation, and surface attachment [96]. DSF is sensed in recipient cells by the RpfC sensor that transmits the signal to the HD-GYP response regulator RpfG to degrade the internal signal molecule cyclic di-GMP [97]. While cell-to-cell signaling was reported to play a significant role in coordinating the expression of virulence genes in multiple pathogenic bacteria, the effect of DSF on the HrpG/HrpX regulon appears to be strain specific. Transcriptome analyses of DSF biosynthesis and signaling mutants in *Xoo* and *Xcci* did not identify the significant regulatory effect on *hrpG, hrpX,* or T3SS genes in artificial media [98,99], and *in planta* in the case of *Xcci* [100]. On the other hand, DSF was reported to play a role in the regulation of the *hrp* regulon in *Xcc*. Analysis of *Xcc rpfC* mutant identified a significant reduction in the expression of *hrpG*, *hrpX,* and their downstream targets [101]. This reduction was also accompanied by reduced HR response in resistant plants and almost complete abolishment of virulence in Chinese radish [101].

## 9. Transcriptional and Post-Transcriptional Regulation of *hrpG* and *hrpX*

Expression, stability, and activity of HrpG and HrpX are dependent on numerous regulatory factors. HrpG and HrpX are negatively or positively controlled by two-component system kinases and response regulators [60,62], ATPases [102], transcriptional regulators [53,63,77,81,84,91,93,103,104,105], small signaling molecules [74,101] and histone-like nucleoid-structuring (H-NS) protein [106,107,108] (Figure 2). However, most studies do not demonstrate direct interactions with HrpG, HrpX, or their promoters and it appears that the regulatory function on this regulon by most of these factors is probably indirect.

**Figure 3:**
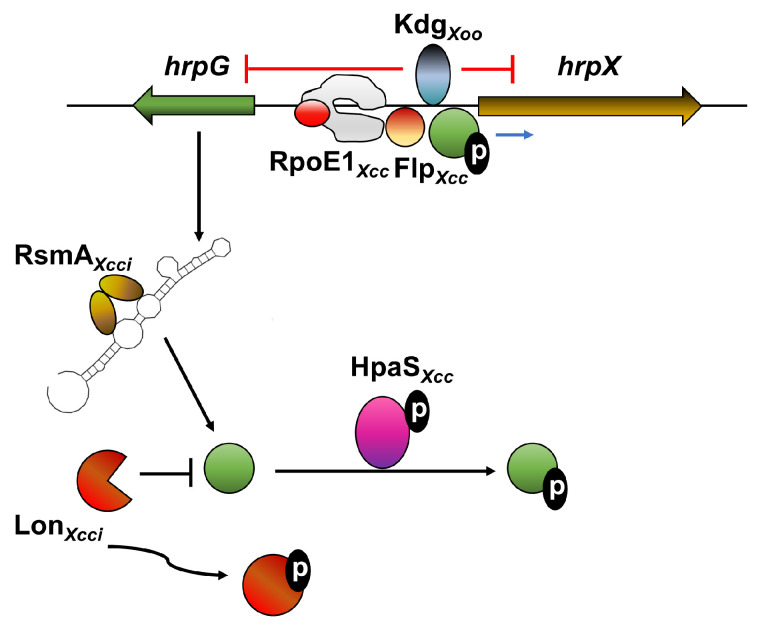
Schematic representation of direct transcriptional and post-transcriptional regulation of HrpG and HrpX. Red “⊣” represents negative transcriptional regulation of *hrpG* and *hrpX* based on transcriptional expression and promoter binding analyses. The Blue arrow represents positive transcriptional regulation of *hrpX* based on transcriptional expression and promoter-binding analyses. Black “⊣” and arrows represent negative and positive post-transcriptional regulation of HrpG, respectively, based on biochemical and functional analyses. Black circled “P” represents protein phosphorylation. Data represent information derived from *X*. *citri* subsp. *citri* (*Xcci*), *X. oryzae* pv. *oryzae* (*Xoo*), and *X. campestris* pv. *campestris* (*Xcc*).

Direct interactions between *hrpG*/*hrpX* and their transcriptional regulators were reported on multiple occasions (Figure 3). Yang et al. demonstrated that the sigma factor RpoE homolog RpoE1 significantly contributes to virulence and *hrp* gene expression in *Xcc* [104] (Figure 2). Further analyses identified that RopE1 is induced in plant-mimicking media XCM1 and controls the expression of *hrpX in vivo* [104]. RpoE1 directly binds to the *hrpX* promoter and promotes *in vitro* transcription of *hrpX*, indicating that RpoE1 directly targets this regulator [104] (Figure 3). Interestingly, RpoE1 is neither affected transcriptionally by HrpG nor affects the expression of *hrpG* itself, indicating that its regulatory function is independent of HrpG [104]. The *Xcc* fis-type transcriptional regulator Flp positively regulates virulence, exoenzyme production, growth in complex media, motility, and *hrp* gene expression [105] (Figure 2). *In vivo* and *in vitro* DNA-protein analyses combined with transcriptional data demonstrate that Flp interacts with the *hrpX* promoter and regulates its expression, suggesting direct regulation of *hrpX* transcription by Flp [105] (Figure 3). GamR, a LysR-type galactose metabolism regulator, was reported to regulate *hrpG* and *hrpX* in plant-mimicking media [77]. The regulator was found to directly bind to the *hrpG/hrpX* intergenic region, suggesting that it is a direct regulator of the two [77] (Figure 2). However, the *gamR* mutant did not display any significant reduction in virulence *in planta* [77]. The transcriptional repressor KdgR of *Xoo* represses the transcription of *hrpG* and *hrpX* by directly binding to their promoter regions [103]. Accordingly, *kdgR* mutant displays enhanced virulence and higher transcriptional expression of *hrpG*, *hrpX,* and *hrp* genes [103] (Figure 2 and Figure 3). Upstream regulation and environmental cues that affect KdgR activity remain unknown.

Post-transcriptional regulation was identified for HrpG in multiple layers. The RNA transcripts of *hrpG* and *hrpD* of *Xcci* are controlled by the CsrA/RsmA RNA-binding protein [109] by binding to the 5′ UTR, which consequently promotes translation and reduces transcript degradation [41] (Figure 3). Deletion mutants of *rsmA/csrA* in *Xcc, Xoo,* and *Xcci* are non-pathogenic, cannot colonize their host or cause HR in resistant plants and display pleiotropic phenotypes including altered colony morphology, EPS production, motility, hydrolase activity, production of DSF, glycogen accumulation, and cell aggregation [41,110,111]. Transcriptome analyses showed that *rsmA/csrA* mutants of all three strains exhibit a significant reduction of transcript accumulation of *hrp* and T3E genes, further supporting that the *hrpG* is under direct control of RsmA/CsrA [41,110,111] (Figure 2). *E. coli* RsmA/CsrA is controlled by antagonistic RNA RsmB/CsrB [112] that does not harbor any known structural homologs in xanthomonads. A potential antagonistic small RNA, RsmU (Figure 2), was recently identified in *Xcc* [113], but environmental cues and upstream regulators of *Xanthomonas* RsmA/CsrA and RsmU have yet to be identified. Intriguingly, flagellar and T4SS assembly genes, which are usually associated with epiphytic fitness, are negatively regulated by *Xanthomonas* RsmA/CsrA [41,114], suggesting that RsmA/CsrA potentially serves as a molecular switch to distinguish between the epiphytic and endophytic lifestyle of the bacteria.

Regulation of HrpG at a posttranslational level was found to be mediated by phosphorylation and protein stability. Similarly to other OmpR-family transcriptional regulators, HrpG harbors a putative phosphorylation site at position D60 [41,42,62]. The ratio of phosphorylated HrpG of *Xcci* is increased when bacteria are incubated in plant-mimicking media compared with rich media [41], supporting that the regulator is activated by phosphorylation. The exact signal and the sensor kinase(s) that promote HrpG phosphorylation were unknown for a significantly long period. Two hybrid studies conducted in *Xcci* and *Xcc* identified two-component sensor kinases that displayed strong interaction with HrpG [62,115]. These sensor kinases, *Xcci* XAC3683 and *Xcc* XC_3670 (HpaS), do not share significant homology or genomic localization with each other. *Xcci* XAC3683 was not subjected to any further study and its significance to the function of HrpG is unknown. The *hpaS* null mutant is deficient in virulence, causes attenuated HR response in resistant plants, and has reduced expression of *hrp* gene in plant-mimicking media and *in planta* [62]. Further analyses identified that HrpG phosphorylation in plant-mimicking media is dependent on HpaS, suggesting that HrpG is directly phosphorylated by HpaS in *Xcc* [62] (Figure 2 and Figure 3).

The protein stability of HrpG is regulated by the Lon protease in *Xcci* [116] (Figure 2). Deletion of *lon* resulted in elevated *hrp* transcript accumulation, hypervirulence in host plants, and stronger HR induction in non-host plants [116]. The observed phenotypes correlate with increased stability of the HrpG protein, indicating that Lon negatively regulates HrpG [116] (Figure 3). *In vitro* and *in vivo* analyses revealed that HrpG is degraded by Lon through recognition of an N-terminal site in HrpG, which promotes its cleavage by the protease [116]. Genetic and biochemical data show that Lon activity is controlled by phosphorylation-mediated deactivation [116]. The environmental cues controlling Lon phosphorylation or the adjusted kinase that phosphorylate Lon are yet to be identified.

## 10. Concluding Remarks

Since its discovery 30 years ago, the *Xanthomonas* T3SS and its regulation by the HrpG/HrpX regulon were subjected to extensive study. While substantial advances were made in understanding virulence regulation in xanthomonads, many questions remain unanswered. Downstream regulation of genes other than the *hrp* T3SS and effector genes by HrpG/HrpX, such as the flagellum assembly genes, needs in-depth study. The upstream regulation of the HrpG/HrpX regulon is not well defined and the direct correlation between metabolic and environmental factors and functional genetic analyses needs further investigation. In addition, although it appears that the HrpG/HrpX regulon is somewhat differentially regulated between *Xanthomonas* spp., it remains unknown which components of the regulatory networks are global, which are species-specific, and what is the biological significance of species-specific regulation to host adaptation and specificity. Further studies dedicated to species-specific regulation of the regulon and dynamic studies of this regulation *in planta* may significantly improve our understanding of bacterial virulence and host specificity.

## Data Availability

Data sharing not applicable.

## Supplementary Materials

The following are available online at https://www.mdpi.com/2076-2607/9/1/187/s1, Figure S1: Multiple DNA sequence alignment of *hrpX*, *hrpG*, and their intragenic regions of *X. euvesicatoria* (*Xeu*) 85–10 strain (genomic region 4996282-4999333), *X*. *citri* subsp. *citri* (*Xcci*) 306 strain (genomic region 1444841-1447887), *X. oryzae* pv. *oryzae* (*Xoo*) KACC10331 strain (genomic region 1411025-1414008), and *X. campestris* pv. *campestris* (*Xcc*) 8004 strain (genomic region 3682705-3685746). Alignment was conducted using Clustal Omega under default settings (https://www.ebi.ac.uk/Tools/msa/clustalo/). The coding regions of *hrpG* are marked in yellow. The coding regions of *hrpX* are marked in green. The Start codons of *hrpG* and *hrpX* are marked in underlined blue. The stop codons of *hrpG* and *hrpX* are marked in underlined pink.
