# Supplementary material for: The HrpG/HrpX Regulon of Xanthomonads—An Insight to the Complexity of Regulation of Virulence Traits in Phytopathogenic Bacteria
Source: Microorganisms. 2021 Jan 16;9(1):187. doi: 10.3390/microorganisms9010187 (PMC7831014; doi:10.3390/microorganisms9010187)
Supplement: Supplementary file 1 [file microorganisms-09-00187-s001.pdf]

```
Xeu          tcagcaggcggctgcgtgatgtgcagggccaagacttgggctaacggccttgctcattgt
Xcci         tcagcaggcggctgcgtgatgtgcagggccaaggcttgggctcacggccttgctcattgt
Xoo          tcagcaggcggctgtgcgatgtgccgggctgagacttaggctgacagccttgctcatcgt
Xcc          tcagcaagctgcggtgcgattgaccgtattggacgtcatgc------ccttggagggtgc
             ******.** ** *.*.***  .* * ......  *.. **      *****   . .*.

Xeu          cgttgcctcggtatcgtgcaacgcgagctccagcttgtagccgtgcgaatagacggttct
Xcci         cgttgcctccgtatcgtgcagcgcgagctccaacttgtagccgtgcgaatagacggttct
Xoo          tgttgcatctttgtcttgcagcgcgagctccaacttgtagccatgcgaatacacggtcct
Xcc          cgccgtgttgccgtccgacagcgccagttcgagcttgtagccatgcgaatacacggtgcg
             .*..*. *.  ..**  .**.*** **.** *.*********.******** ***** *

Xeu          gatgcgcaccgcgctgctgtcaccgctcagctgcagtttcttgcgcaacttgtagatgtg
Xcci         gatgcgcactgcgctgctgtcactgctcagctgcagtttcttgcgcaacttgtagatgtg
Xoo          gatgcgcactgcgccgttgtgattgccgagctgcagtttcttgcgcagcttgtagatatg
Xcc          aatgcgcactgcgccactgtggttgcacagccgcagtttcttgcgcagcttgtagatgtg
             .********.****...*** ...**  ***.***************.*********.**

Xeu          ctgctccatggtgcggtcggtgaattcggtatggctgccccagaccgccttggccagctg
Xcci         ctgctccatggtgcggtcggtgaattcggtgtggctgccccagacggccttggccagttg
Xoo          ctgctccatggtgcggtcggtgaattcggtgtggctgccccagaccgccttggccagctg
Xcc          ctgttccatggtgcggtcggtgaactcggtatggctaccccagactgccttggccagctg
             ***.********************.*****.*****.******** ***********.**

Xeu          gcaacgccggaagcacacgccggggctggaaaagagcagccaggcaatcgagaactcgcg
Xcci         gcaacgccggaaacacacgccggggctggaaaagagcagccaggcaatcgagaactcgcg
Xoo          gcaacgccggaagcacacgcctgggctggaaaatagcagccaggcaatcgaaaactcgcg
Xcc          gcagcggcggaagcacacgcccggattcgaaaacagcagccaggcgatggagaactcgcg
             ***.** *****.******** **..* ***** ***********.** **.********

Xeu          tgcggtcaacgcgatcggcttgccttccaggtagacggtattttcatcgcggatcagctt
Xcci         tgcggtcaatacgatcggcttgccgtccagataaacggtattttcatcgcggatcaaccg
Xoo          tgcggtcagcacgatcggcttgccatgcagatacacggtattttcgtcgcggagcaactg
Xcc          cgcggtcaaggcaatcgcggtttcgtgcaggtacaccgtgttcttctcgcggagcagctg
             .*******. .*.****   * .* * ***.** ** **.**.*. ******* **.*.

Xeu          gtagggacccacgctcaggtgctgcgtctccgggcaggcatgcgccaccggcgatatcgc
Xcci         gtatggaccaacgctcaggtgctgcgtctccgggcaggcatgcgccaccggcgatatcgc
Xoo          gtatggaccaacgctgagttgctgcgtttccgggcaggcatgcgccaccggcgatagcgc
Xcc          atacgggcccaccttgatctgctgggcctggatgcaggtggctgccatcggccaaatggc
             .** **.** ** .* *  ***** *..*  . *****..  .****.**** * *  **

Xeu          cagggccgcgcgcacgtgcaactcatgcgaattgaaaggcagcgcaaggacctcctgcgc
Xcci         cagcgccgcgcgcacgtgtaactcgtgcgaattgaaaggcagcgcaaggacttcctgcgc
Xoo          cagggccgcgcgtacctgcaattcgtgcggattgaaggggagcgcaagcacctcctgcgc
Xcc          gagcgatgcgcgcacatgcaactcgtgtgagttgactggcaggctgagcacttcgtgggc
              ** * .*****.** **.**.**.**.*..****  ** **  ..** **.** ** **

Xeu          gcctgcgcgataccaggccagaatgttgtcggcgcaatcgaaacggccaagcacgatcag
Xcci         gcctgcgcgataccaggccagaatgttgtctgcgcaatcgaaacgtccaagcacgatcaa
Xoo          gcccgcgcgataccagtccaggatgtcgttggcgcagtcgaaacgcccgagcacgatcag
Xcc          gccggcgcgataccaggccagcaagtcgtcggggcactcgaaacggcccagcacaatcag
             *** ************ **** * **.**. * *** ******** ** *****.****.

Xeu          cggcgtgggctggccgctatggcagcgctgccaggccagcagtgaactgtcatcggcggc
Xcci         cggggtgggctggccgctatggcagcgctgccatgccagcagtgaactgtcatcggcggc
Xoo          cggcgtgggatgaccgctatggcagcgctgccaggccagcagcgagctttcgtcggaggc
```

```
Xcc             tggggtggggtgaccactgtggcagcgctgccaggcgagcagggaactgtcgtccgatgc
                .** ***** **.**.**.************** ** ***** **.** **.** *  **

Xeu             gacacagctggcatcgaagatcagcagctcgcacggcgagtgccgcagcgaacgcaggag
Xcci            gacatagctggcatcgaagatcagcagctcgcacggcgaatgccgcagcgtacgcaaaag
Xoo             gacgcaactggcgtcgaagaccagtaactcgcacggcgaatgccgcaacgaacgcaggag
Xcc             cacgcaatgggcatcgaaaaccagaagctcgcacggcgagtggcgcaggcagcgcagcaa
                 **..*.. ***.*****.*.*** *.************.** ****.   .****. *.

Xeu             ctccagttcatcggaaaacgtcgagacgttgcgtgcgagcggtgcaaggctggcgttgac
Xcci            ctccagttcatcggaaaacgtcgagacattgcgcgcaagcggtgcgaggctggcgttgac
Xoo             ctccagttcatcggagaacaccgagacattgcgcgatagcggtgcgatgctggcattgac
Xcc             ttcgagttcgtctgaaaagatggtgacctctgggaccagagtcgccaggctggcgttgat
                .** *****.** **.** .. * *** *.  * .  ** * .** * ******.****.

Xeu             ctgcgaggtcaggcgcgcgtcctgcgtcaacaggaacaccgatccggcattggaggggga
Xcci            ctgcgagaccaggcgcgcatcctgcgtcaacaggaacaccgatccggcgttgggggagaga
Xoo             ctgcgaggccaggcgggcgtcctgcgtcaacagaaacgccgatccgtgtgcaagggggca
Xcc             ctgtgatgccagccgcgcgtcctgcgtcagtacgaatgccgatccttggcggtcattcgc
                ***.** ..*** ** **.**********..* .**..*******...   . .... ..

Xeu             gtggtcgttcattcaggcggccttcgcgtgtcggcgtacaagccacgcatgggacgggcg
Xcci            gtggtcgttcatttaggcggccttcgcgtgtcggcgtagaagccacgcatggggcgggcg
Xoo             agggtcgttcat-caggtggacatcccgtggccgcgcgcaaaccacgcgtgggccggacg
Xcc             agcggcgtccatcactcgcgcgc-------------------ccacgccggcggtacggg
                ...*...*****  ... .* .                    ******  * * .. . *

Xeu             ccggtgcgggtcggccaatgccaggacaaaatccatcttcaaag---atcgctgcatcgt
Xcci            ccggtgctggtcggccaatgccaggacaaaatccatcttcaaag---atcgctgcatcgt
Xoo             ccgacgcctggcgcccgacgccaggacaaaacccacagggagag---atcgatgcatcgt
Xcc             cgcagcccagaccatggaactgagcacaacaggcttactaaaggtatccaggcacatagc
                *  .  *  * *  . .*  . ** **** *  * .    *..*    . * ..*** *.

Xeu             cggaccagtcggttggcgggggagagtccag-tggagctggacgaatggtatgtaccatt
Xcci            cggaccagtcggttggcgggggagagtccag-cggagctggacgaatggtatgtaccatt
Xoo             cggaccagttggttgtcgggggagcgtccgg-cagcgccggaccaatgctatttaccatt
Xcc             catatcactcgaccgcttcgggggatctgacctgcgtcagacgaatggtatgtaccatt
                *. *.** *.*...* .  ***.* .**... . * *...*** **** *** *******

Xeu             gcagagaccaaaagtttcacgcttgcgtacgcgtcttcggatttgtcga--catcggctt
Xcci            gccgggaccaaaagtttcacacttgcgtacgcggcttcggatttgtcgc--cagcggctt
Xoo             gcaacgaggaaaagtttcacgcttgcgaacgcttcttcgggtttgtgag--ggttggctt
Xcc             gcggaggcgaaaagttt--cgttgcctagcgcgaattccgatcagcggagtcatccgctt
                ** . *.  ********  *..*  *  .***   *** *.*. *. .    . . ****

Xeu             taactcgcgctcatttagacgtaacagagctcttacatagcgggcatgtctgggacgcat
Xcci            tagctcgcgctcatttagacgtaacagagctcttacatagcgggcatgtctggtccgaat
Xoo             taactcgcgctcatcttggcgtaacggagctcttacataacgggcatgtgggcgtcgg--
Xcc             tagcgggtggtcagttagccgcaacacagctcttactctctagttatgcaatcgtcgtct
                **.*  *.* *** .* * **.***. ********* .  ..* .***.      **

Xeu             g-----------------------------------caattgcgccatgcgcgtcatgca
Xcci            g-----------------------------------caatcgctacatgca-gtcatgca
Xoo             ----------------------------------------cactgcgcaca---------
Xcc             gacgcatagggctggttggggcgttttaattcggtcgggttgctgcacaca---------
                                                        ..*  *...*.
```

```
Xeu             gtagcgagtaagcatggaccgacgcgcgcgggcagaggcatccggttgccgaggtctaga
Xcci            gca------aggcatggaccgacacgcgtgaccagagacatcggtccgccgaggtctaga
Xoo             --------------------------------------------gccgccaaggtataga
Xcc             ----------------aaaccccatgctggagcggcggcatgccaacgtgcaggtgtaga
                                                              .*.  **** ****

Xeu             ccaatctattgcgattggtcgtgggatcgcgcgcg-gctggaataggtcag-cagcgatt
Xcci            ccaacctattgcgattggtcgtgtgatcgcgcgtg-actggaataggtcgg-cagtgatc
Xoo             ccaatctattgcgattcgtagtgcaatcacgcgcattctggaatacatcggtcaacaatt
Xcc             ccaaggtatagagattt-tgctgactccctgcgcgagaaagaatatctcga---gggact
                ****  *** * ****  *  **   .* .***..    .*****  **..   . .*..

Xeu             ttcct------tcgtggcatgcccgcttctcgcgatcaaaaacgcttcggc---------
Xcci            tgccc------tcgtgtcatgtctgcttctctcgatcaaatgcgcttgggc---------
Xoo             cggct------gcgcgctaggtctgccgctcatcattaagccagtgtcggc---------
Xcc             tgactatcgcgccgctgaacaaaatgctcttgtaattacaggtggaccggcgaatcgccg
                .  *.       **.   * .     . **. . **.* .   *  . ***

Xeu             -------gtacgtgtccgtacagtcacggtgcatcgtgttgaacatcgccgatgaccatc
Xcci            -------atacgtgtccgtacagtcatcgggcatcgccttggtcatcgccgacagccatc
Xoo             -------gtgtgtgttcacaaaattgcag----------tggctgcggccaataccggtc
Xcc             gaaaacagcgtgtgccctcattgcactgg---------------------attaacggcc
                       ....***..* .*  ..  . *                     . .. * ..*

Xeu             aatgcgcatggaagcggcgcgttcgcagacgttgtccgactgtgcc-tggggattggcgt
Xcci            aatgcgcatggacgcagcgcgttcgcagacgttgtccgactgtgccttggatattggcgc
Xoo             aatggctgtggaagcggcgcgttcgcagacgttgtccgactacctg-cgcgcattggcac
Xcc             atttgacattgagcgcgagcgatcgcagatgtttagcgcgccttcg-cgcatctgcgtgc
                * *   ..* **    * *** *******.***   **  . . .  .* .  *  *...

Xeu             agaccggcgatatcaatactcaaccggatggcgcgaaatgtctggcg-tacgacaatg--
Xcci            agaccggcgacatcaatactcaaccggatggcgcgaaatgtctggcgttacgacgacg--
Xoo             agaccaatggcatcgatatccggccggatggcatgaaatgtcttgtg-cacgacactg--
Xcc             cgaacaccg-catccgtacgc---------ccacgcatcgtcctgtgatgcaccgtcaag
                 ** *. .* .*** .**. *          *..* * .***. *.* ..*. *. ..

Xeu             ------------------------ccaacgatgtgcaaatgcgctttgcgtcgggagaaa
Xcci            ------------------------tcgacggtgtgcaactgcgctttgcgccgcgaaaaa
Xoo             ------------------------ctggaggtatgcaaaaactcactgcgtctcgcg-aa
Xcc             atacgccggcgcatcgtctggccttcgtcgctacgcaggtgcgggatccacttcgca---
                                        ...  * *..***.  .*    * *...  * .

Xeu             acgcatcacatggttgtttg----acagagtgagcttttttgattttttcaatggctttc
Xcci            acgcatcacatggatgtttg----acagagtgagcttttttgattttttcgatggctttc
Xoo             acgcacgacgtgctcgtttg-agaacagagtgaggttttttgattttttcagtctcttca
Xcc             ---------------gtttgcaagacagagtgagaatttttgaatttctagctgcttcga
                               *****    **********  ******* ***.* . *  .*.

Xeu             agagggaaattctattggatccgctgcatacaatcgtttgcgccagcgagttcggcgcct
Xcci            agagagaaattctattggatccgctgcatacaatcgtttgcgccagcgagttaggcgcct
Xoo             gttgacaaattccattggatccgctgcatacaatcgtgtgcgccggcgagctcggcgatt
Xcc             agtgagaaattctattggatccgctgcatacaatcgtttgcgcccaccacaacggcgcct
                .  *. ******.************************ ****** .* *    **** .*

Xeu             gttgt--cttttgctccgccccccaaagagagagaccggcatgatcctttccacctactt
Xcci            gttgt--cttttgctccgccccccaaagagagagaccggcatgatcctttccacttactt
Xoo             gttgt--cttttgctccgcccccc-aagagagagaccggcatgatcctttcgacctactt
```

```
Xcc       tttgttgtttttgctcctcccctt---cagagagcccggcatgatcctttcgacctactt
           ****  .********* ****..    ****** **************** **.*****

Xeu       tgcagcgatctctgcgttgtcatacgcagaacgtcttcctacctatacgagcaggatgct
Xcci      tgcagcgatctctgcgttgtcctacgcagaacgtcttcctacgtatacgagcaggatgct
Xoo       tgcagcgatctctgcgttgtcttacgcagaacgtcttcctacctatacgagcaggatgct
Xcc       cgcagcgatctctgcgttgtcttacgcagaccgtcttccgatctatacgagcaggatgct
          .******************** ******** ******** *. *****************

Xeu       ggttggtgcttggccacagggactgcaacatctccaacagcgacgcgacggacagggcgc
Xcci      ggttggtgcttggccacagggactgcaacatctccaacagcgacgcgacggacagggcgc
Xoo       ggttggtgcttggccgcagggactgcaacaccttcaacagcgacgcgacgggcagggtgc
Xcc       tgttggtgcttggccacagggtctgcagcacctccagcaacgtcgcgaggggcaggcagc
           **************.***** *****.**.**.**.**.** ***** **.****  **

Xeu       agcggatggcgccgacgatgaggtcagcttgttcggtgccagcggcgatgcgttgctgat
Xcci      tgtggatggcgccgatgatgaggtcagtttgttcggtgccagcggcgatgcgttgctgat
Xoo       agccgatggcgccgacgatgaggtcagcctgtttggtgccagcggcgatgcgctgctgat
Xcc       tccggatggcagcgacgacgaagtcgcattgctgggcgccagcgccgatgccttgttgat
            . ******. ***.**.**.***.  .**.* **.******* ****** .**.****

Xeu       cctggaatatcaggaagaggccgaagatgcgtatcggcaggccttgaaggccatgcgcgg
Xcci      cctggaatatcaggaagaggccgaagatgcgtatcggcaggccttgaaggcaatgcgcgg
Xoo       cctggaatatcaagaagaggccgaagacgcgtatcggcaggctttgaaagccatgcgcgg
Xcc       tctggagtatcaggaagaggcagaggatgcctatcggcaggcgtccaaggcgatgcgtgg
          .*****.*****.******** **.**.** *********** *. **.** *****.**

Xeu       gcatcagcgccaattgcgcctgctgtcgtgccggaacaccgcctggttgatgctgagaca
Xcci      gcatcagcgccaattgcgcctgctgtcgtgcaggaacaccgcctggttgatgctgagcca
Xoo       gcagcagcgccaattgcgtttgctgtcgtgccggaataccgcctggttgatgctgagcca
Xcc       gcaccagcggcagctgcgcctgctgtcgtgccgcaacaccgcgtggctgatgttgagcca
          *** ***** **..****..*********** * **.***** ***.*****.**** **

Xeu       gcggcgcctgagcgcggcgttgaattgcttcgcgcagttggcaatggaccgcgaaacgcc
Xcci      gcggcgcctgagcgcggcgttgaattgctttgcgcagttggcaatggaccgcgaaacgtc
Xoo       gcgacgcctgagtgcggcattgaactgctttgcgcagctggcgatggatcgcgaaacgcc
Xcc       gcgccggctgagtgcagcgctcaattgctttgcgcaactggccatggaccgcgatacccc
          *** ** *****.**.**..* **.*****.*****..**** *****.***** ** .*

Xeu       gcccagcctgtgcggcgagagcctgttgggcaaatcgctcacccacttccatctgggcca
Xcci      gcccagcctgtgcggcgagagcctgctgggcaagtcgctgacccacttccatctcggcca
Xoo       ccccagcctgtgcggcgagagcctgctgggcaaggcactgacccactttcatctgggcca
Xcc       ggcggtgctgtgtggcgagagcctgctcggcaaggcgctgacccacttccacctgggcca
            * .  *****.************.* *****. *.** ********.**.** *****

Xeu       gagcacgttggccttacagaccctggagcgtgcccgcgaggtgctggccgacctggagag
Xcci      gagtacgttggccttgcagaccctggagcgcgccagcgaggtgctggccgacctggagag
Xoo       gagcacgctggccttgcagaccctggagcgcgccaacgaggtgctggccgagctggagag
Xcc       gagcaccctggcactgcagacgctggaacgcgcgcaagaggtggtttccgagctggagct
          ***.** .**** .*.***** *****.**.**  . ****** *  **** ******

Xeu       cggcgcgtcggactggctgcggattgccactgcgctgcgtctggacatgattgcgcagct
Xcci      cggggcgtcggaatggctgcggatcgccaatgcgctgcgtctggacatgattgcgcagct
Xoo       tggtgcttccgattggctgcgtgtcgccaccgcgctgcggctggacatgatcgcgcagct
Xcc       cggtgcggcggactggacgcgtgtggtcggcgcgctgcgtctggacctgatcgcccagct
          .** **  * ** *** .*** .* *.*. .******** ****** ****.** *****
```

```
Xeu                 gcgtatccgtcgctccgaccgcatgagcgaccatgtgttctggcaagccacgctgcgcca
Xcci                gcgtatccgtcgctccgatcgcatgagcgaccatgtgttctggcaagccacgctgcgcca
Xoo                 gcgcatccgccgctccgatcgcatggtcgaccatgtgttctggcaggccacgctgcgcca
Xcc                 gcggatccggcgcggcgagcgcatgagcgaccacgtgttctggcaggccacgctgcgcca
                    *** ***** ***   *** ******. ******.***********.*************

Xeu                 ggaagatgccacgcatgccttcgagcccagcgacctgcgcgcctgcatcgccgatctggc
Xcci                ggaagatgccgcgcatgccttcgagcccagcgacctgcgcgcctgcatcgccgatctggc
Xoo                 ggaagatgccgcgcatgccttcgagcccagcgatttacgcgcatgcatcgccgatctggc
Xcc                 ggaagatgccgcgcataccttcgaggccagcgagctgcgtgcctgcatcgccgatctgtc
                    **********.*****.******** ******* .*.**.** *************** *

Xeu                 agagagcatgccggtgcttgcgcagcgcatgcggcatgtacgcagcctgttgcgtattgc
Xcci                agaaagcatgccggtgctggcgcagcgcatgcggcatgtgcgtagcctgctgcgtattgc
Xoo                 cgagagcatgccggtgctggcgcagcgcatgcggcatgtgcgcaacctgctgcggatcgt
Xcc                 cgagagcatgccggtgctggcgcaacgcatgcgccatgtgcgcaatctgctgcgcattgc
                     **.************** *****.******** *****.**.*..***.**** **.*.

Xeu                 cggcggcgacaccttcggcttcgacgcgcagatcgacgcattgccggccgcagccaccgg
Xcci                cggtggcgacaccttcggcttcgacgcgcagatcgacgcattgccggccgcggccaccgg
Xoo                 cggcggcgataccttcggcttcgatgcgcagatcgatgcgttgccggctgcggccaccgg
Xcc                 tggtggagacatcttcggcttcgacgcgcagatcgatgccttgcccgcagcggccaccgg
                    .**.** **.*.************.***********.** ***** ** **.********

Xeu                 ctgcccgccgtgtgcgcgtcaggacgcgcaggtggaactggcgctggcggcattggcggt
Xcci                ctgcccgccgtgcgcgcgtcaggacgcgcaggtggagctggcgctggcggcattggcggt
Xoo                 ctgcccgccgtgtgcgcgtcaggacgcacaggtggaactggcgcttgccgcattggcggt
Xcc                 ttgcccgccatgcgcgcggcaggatgcgcaagtggagctggcgctggccgcattggcggt
                    .********.**.***** *****.**.**.*****.******** ** ***********

Xeu                 gggccgtgccgatctggccgagcgtgcgctggccggtgccggccgacatcatgcgccgcg
Xcci                gggccgtgccgatcttgccgagcgtgcgctggccggcgccggtcggcatcatgcgccgcg
Xoo                 ggggcgtgccgatctggccgagcgcgcgctggccggtgccggccggcatcatgcgccgcg
Xcc                 tgggcgtgcggacctggccgaacgcgcgctggccggtgccgggcgccatcacgcgccgcg
                     ** ***** **.** *****.**.***********.***** ** *****.********

Xeu                 ctggaacctggagttcgagtactgccaggccaagatcagccaggcgatggggcgcaccga
Xcci                ctggaatctggagttcgagtactgccaggccaagatcagccaggcgatggggcgcaccga
Xoo                 ctggaatctggagttcgaatactgccaggccaagatcagtcaggcgctgggacgcacgga
Xcc                 ctggaacctggagttcgagtattgccaggccaagatcagccaggcactgggacgtgccga
                    ******.***********.**.****************.*****. ****.**..* **

Xeu                 acaagcgttgctgctctacaaccgctacgcgctgggcgcggtgcagtgcctgcgcagcga
Xcci                acaagcgttactgctctacaaccgctacgcgctggacgcggtgcagtgtctgcgcagcga
Xoo                 acaagcgttgttgctctacaaccgctatgcgctggacgcggtgcaatgcctgcgtagcga
Xcc                 gcaagccttgctgctctacaaccgctacgccctggggctgtgcaatgcctgcgcagcga
                    .***** **..***************.** ****. ** *****.**.*****.*****

Xeu                 agtgcaggcaccgcgcttgctggaaagcggcaccccggcgcacgtcagcgacgatatctc
Xcci                agtgcaagcgccgcgcttgctggaaagcggcacaccggcccacgtcagcgacgatatctc
Xoo                 agtgcaggcaccgcgcttgctggaaagcggtaccccagcgcacgtctccgacgatatttc
Xcc                 ggtccaggcgccgcggttgctggaaagcggcacaccggcgcatgtcagcgatgacatttc
                    .** **.**.***** *************.** **.** **.***   ***.**.**.**

Xeu                 cgcccggttgccggccaagtatcggcgcgcctacagctacatgatcaccaatgcgcatcg
Xcci                cgcccggttgccggccaagtatcgacgcgcctacagctacatgatcaccaatgcgcatcg
Xoo                 cgcccgcttgccggccaaatatcggcgtgcgtatagctacatgatcaccaacgcgcaccg
```

```
Xcc                 cgcccgcctgccggccaagtaccggcgtgcctacgcgtacatgatcaccaatgcccatcg
                    ****** .**********.**.**.**.** **..  **************.** **.**

Xeu                 ctcggatctgtcgatcaacgaagtggccgcacagatcggggtcaccggcgcgcgctgca
Xcci                ctcggatctgtcgatcaacgaagtggccgcacagatcggagtcaccggcgcgcgctgca
Xoo                 ctcggatctgtcgatcaatgaagtggccgcacagattggggtgaccggcgcgcactgca
Xcc                 atcggacctgtcgatcaacgaggtcgccgcgcagatcggggtgaccggcgcgcgctgca
                     *****.***********.**.** *****.*****.**.** ***********.*****

Xeu                 gcaagcgttcaagtcggctaccgggctatcgccgacgcaggtgctgcgccgctaccggat
Xcci                gcaagccttcaagtcggctaccgggctatcgccgacgcaggtgctgcgccgttaccggat
Xoo                 gcaggcgttcaagtcggccaccgggctgtcgccgacccaggtgctgcgccgttaccgcat
Xcc                 gcaggccttcaaatcagcgaccggcttgtcgccgacccaggtgctgcgccggtatcgcat
                    ***.** *****.**.** ***** .*.******** ************** **.** **

Xeu                 gcaaagcatccgcgacgaactgctggccgaaggcggctgcggcagtgtgttgcaggcggc
Xcci                gcaaagcatccgcgacgaactgctggccgaaggcggctgcggcagtgtgttgcaggcggc
Xoo                 gcagagcatccgcgacgaactgctggccgaaggcggctgcggcagcgtgttgcaagcggc
Xcc                 gcagggcatccgcgatgaactgttggccgatggcggttgtggcagcgtgctgcaggcggc
                    ***..**********.******.******* *****.**.*****.***.****.*****

Xeu                 cagtcgctggggcgtcggcagccgttcggcgttggccaagggctatcgccagcacttcaa
Xcci                cagtcgttggggcgtcggtagtcgttcggcgttggccaagggctatcgccagcacttcaa
Xoo                 cagccgctggggtgtgggcagccgttcggcattggccaagggctatcgccagcacttcaa
Xcc                 gagccgctggggtgtgggcagtcgctcggcgctggccaagggctatcgccagcatttcaa
                     **.**.*****.** **.**.**.*****..**********************.*****

Xeu                 tgaggcgccgatggagaccctgcagcggtaa
Xcci                tgaagcgccgatggagaccttgcagcggtaa
Xoo                 cgaagcaccgatggaaaccttgcaacggtaa
Xcc                 tgaagcgccgatggaaaccctgcaacgctaa
                    .**.**.********.***.****.** ***
```

Fig S1. Multiple DNA sequence alignment of *hrpX*, *hrpG* and their intragenic regions of *X. euvesicatoria* (*Xeu*) 85-10 strain (genomic region 4996282-4999333), *X. citri* pv. *citri* (*Xcci*) 306 strain (genomic region 1444841-1447887), *X. oryzae* pv. *oryzae* (*Xoo*) KACC10331 strain (genomic region 1411025-1414008) and *X. campestris* pv. *campestris* (*Xcc*) 8004 strain (genomic region 3682705-3685746). Alignment was conducted using Clustal Omega under default settings (https://www.ebi.ac.uk/Tools/msa/clustalo/). The coding regions of *hrpG* are marked in yellow. The coding regions of *hrpX* are marked in green. The Start codons of *hrpG* and *hrpX* are marked in underlined blue. The stop codons of *hrpG* and *hrpX* are marked in underlined pink.
